# The Role of Character Strengths in Depression: A Structural Equation Model

**DOI:** 10.3389/fpsyg.2018.01609

**Published:** 2018-09-06

**Authors:** Ata Tehranchi, Hamid T. Neshat Doost, Shole Amiri, Michael J. Power

**Affiliations:** ^1^Psychology, University of Isfahan, Isfahan, Iran; ^2^Psychology, National University of Singapore, Singapore, Singapore

**Keywords:** character strengths, depression, dysfunctional attitudes, happiness, negative affect

## Abstract

The main aim of present study was to develop a model that specifies the predictive effects of some character strengths in depression. Two hundred individuals with major depression were recruited from clinical psychology centers. Participants completed a battery of questionnaires measuring dysfunctional attitudes, basic emotions, character strengths, and depression. Seven character strengths of critical thinking, emotional intelligence, gratitude, forgiveness, hope, spirituality, and zest were selected to measure the latent variable of character strengths. Structural equation modeling was used to analyze the data. Normed chi-square, comparative fit index, incremental fit index, and other indices demonstrated an adequate fit for the model suggesting that character strengths had an indirect effect on depression through the mediation of dysfunctional attitudes, negative affect, and happiness. Character strengths had negative effects on dysfunctional attitudes and positive effects on happiness. The findings of present study have implications for practitioners and researchers to develop an integrative model for the treatment of depression.

## Introduction

Depression is one of the most common and debilitating mental disorders (Ross et al., 1997). It is characterized by symptoms such as sadness, loss of pleasure, irritable mood, and feelings of worthlessness (DSM-5; American Psychiatric Association., 2013). The etiology of depression is complex and no single cause can account for it. People with depression typically experience cognitive distortions and the role of dysfunctional attitudes and beliefs is emphasized in the cognitive psychopathology of depression (Miranda and Persons, 1988; Teasdale, 1988; Meyer et al., 2003). Although the decision as to whether an attitude is dysfunctional or not is dependent on the context, many studies have demonstrated that these attitudes are considered as dysfunctional across different cultures such as Turkish and Iranian (Sahin and Sahin, 1992; Ebrahimi, 2009). It is assumed that dysfunctional attitudes can lead to negative emotions and dysfunctional behaviors. Nevertheless, some studies have argued that the cognitive psychopathology model of emotion is inadequate (e.g., Le Doux, 1996; Zajonc, 2000). For example, it has been shown that emotions can precede cognition and that they can also have an integral contribution to information processing (Forgas, 2000; Greenberg, 2002).

Given such findings, other researchers have proposed etiological models of depression that have focused more on emotions (Greenberg and Watson, 2006; Power, 2010). It has been shown that individuals with depression experience high levels of sadness, shame, and anxiety that intensely affect their self-organization and mode of processing (Greenberg and Watson, 2006). Some emotions are experienced as positive when people are involved in positive antecedent situations (e.g., meeting friends) and some are considered as negative when people encounter with negative antecedent situations (Lim, 2016). It has been shown that similar emotions are experienced in similar situations across cultures (Lim, 2016). Thus, the terms of negative and positive emotions can be used across different studies and populations.

Negative emotions can be incorporated into a construct called negative affect. Depression is related to negative affect, but it is also different (Danhauer et al., 2013). Individuals may experience negative affect whether or not they are depressed (Danhauer et al., 2013). Furthermore, depressed individuals tend to experience non-mood depressive symptoms (i.e., somatic, vegetative) as well as negative affect (Danhauer et al., 2013). Thus, negative affect and depression are correlated but they are not interchangeable constructs. Negative emotions such as shame or guilt, fear or anxiety, sadness, and anger are considered to be key contributors to the development of depression (Greenberg and Watson, 2006). Power and Dalgleish (2008) proposed an emotion-focused model for the etiology of depression. Based on this model, coupling and pairing of sadness and disgust as two basic emotions lead to depression (Power, 2010).

Cultural factors need to be considered when studying the role of emotions in depression. For example, the findings of a study conducted on 600 immigrant Asian-, European-, and Asian American college students revealed significant relationships between positive emotions and depressive symptoms among European Americans and Asian Americans but not Asian immigrants in America (Leu et al., 2011). Importantly, the association between negative emotions and depressive symptoms was significant among all three groups (Leu et al., 2011). To our knowledge, the role of basic emotions in depression has not been studied in an Iranian population.

Vulnerabilities and shortcomings of clients are emphasized in cognitive and emotion-focused psychopathologies of depression. However, the etiology of depression can also be viewed as a lack or excess of capabilities and strengths (Rashid, 2014). Positive Psychotherapy (PPT) as a recent approach for the treatment of depression is based on the assumption that each individual has inherent capacities for growth, fulfillment, and wellbeing (Seligman, 2012; Rashid, 2014). This approach argues that psychopathology occurs when a client's capacities are thwarted by psychological and sociocultural factors (Rashid, 2014). Finding and cultivation of capabilities are based on Peterson and Seligman's work on character strengths (Peterson and Seligman, 2004). There are 24 character strengths classified under six virtues (Peterson and Seligman, 2004). Disabato et al. (2014) examined whether these “strengths” predicted the onset of depression or whether depression lead to a decreases in these strengths. Utilizing longitudinal methods the researchers found that character strengths reduced depression symptoms, but depression did not significantly reduce character strengths (Disabato et al., 2014). Similarly, another study which investigated the role of character strengths in subjective wellbeing among adolescents from a general population found that other-directed character strengths predicted fewer symptoms of depression (Gillham et al., 2011). It has also been demonstrated that the overuse or underuse of character strengths is significantly associated with higher depression, less flourishing, and less life satisfaction in the general population (Freidlin et al., 2017). Specifically, the underuse of character strengths had a stronger relationship with depression rather than did overuse (Freidlin et al., 2017). Psychotherapists in PPT help clients to identify, understand and cultivate their inherent character strengths (Rashid, 2014).

Given the findings reviewed above, some vulnerabilities and lack of some capabilities are assumed to cause depression. In clinical practice, many researchers have proposed the integration of clinical and positive psychology elements to consider both negative and positive aspects of a clients' life (Wong, 2010; Wood and Tarrier, 2010; Bannink, 2014; Chao, 2015). It seems that considering both capabilities and vulnerabilities can result in a more comprehensive formulation of depression. Such an understanding can help practitioners and researchers to be better able to predict, explain, and conceptualize depression. To our knowledge, no study has been carried out to examine relationship of vulnerabilities and character strengths with depression. Furthermore, the relationship between character strengths, vulnerabilities (negative emotions, dysfunctional attitudes), positive emotion, and depression has also not widely been investigated.

It can be hypothesized that character strengths have certain effects on negative emotions and dysfunctional attitudes. In order to examine this hypothesis, some character strengths were selected in the present study. The selection criteria for character strengths was based on either a theoretical or empirical relationship between the strengths and depressive symptoms such as despair and anhedonia. Similarly, selection criteria was also based on the theoretical or empirical relationship between strengths and specific treatment factors including emotion management and critical thinking. Among the strengths classified under the virtue of wisdom (Peterson and Seligman, 2004), critical thinking can be theoretically associated with the cognitive psychopathology of depression. Individuals with the character strength of critical thinking are expected to have lower dysfunctional attitudes. Previous research has also demonstrated that critical thinking was significantly correlated with affective control (Esmaeili and Bagheri, 2015). The second category of strengths is classified under the virtue of courage. Among these, vitality was selected. Lack of zest (vitality) can be related to anhedonia, one of the most predominant symptoms of depression. Interpersonal strengths that involve tending and debriefing others are classified under the virtue of humanity (Peterson and Seligman, 2004). Among them, emotional intelligence was selected as deficits in the character strength of emotional intelligence can increase negative emotions. Civic strengths underlie healthy community life but these character strengths were not selected from as they did not meet the selection criteria. The strengths that protect against excess are classified under the virtue of temperance. Forgiveness was selected among them as it was assumed that individuals with forgiveness can manage negative emotions such as anger and disgust properly. Previous research has also demonstrated that the ability to manage and repair emotions was associated with a greater disposition toward forgiveness (Hodgson and Wertheim, 2007). The character strength of self-regulation was not selected as it is included in emotional intelligence (Salovey and Mayer, 1990). The three character strengths of gratitude, hope, and spirituality were selected from the strengths classified under the virtue of transcendence. These strengths forge connections to the larger universe and provide meaning. Hope met the selection criteria as lack of hope or despair is a common symptom of depression. Gratitude mitigates negative experiences including resentment, envy, and regret (Fredrickson, 2004). It has also been demonstrated that individuals higher in spirituality may possess higher levels of hope, optimism, gratitude, and compassion (Park, 2007) and have lower levels of negative affect (Koenig et al., 2001). In general, many studies have indicated that the strengths of gratitude, forgiveness of oneself, spirituality, hope, emotional intelligence, and critical thinking are significantly and negatively correlated with depression (Doolittle and Farrell, 2004; Downey et al., 2008; Toussaint et al., 2008; Wood et al., 2008; Thimm et al., 2013; Esmaeili and Bagheri, 2015; Luna and MacMillan, 2015). Character strengths can also increase positive emotions. Previous studies have demonstrated that most character strengths show a positive correlation with positive affect (Littman-Ovada and Lavy, 2012; Azañedoa et al., 2014; Martínez-Martí and Ruch, 2014). Therefore, we selected the seven character strengths of hope, critical thinking, emotional intelligence, vitality, forgiveness, gratitude, hope, and spirituality in the present study. However, we do not claim that this list of strengths is comprehensive as they only represent a selection of the most important strengths that may buffer people experiencing depression.

One study has investigated the role of some character strengths' (classified under the virtue of transcendence) relationship to depression among individuals with and without major depression (Huta and Hawley, 2010). The findings demonstrated that character strengths were related to depressive scores among individuals without major depression (Huta and Hawley, 2010). Interestingly, there was no significant correlation found between the character strengths and depression scores among individuals with major depression (Huta and Hawley, 2010). However, they found that pre-treatment character strength predicted post-treatment recovery (Huta and Hawley, 2010). This suggests that the role of character strengths in depression might be through indirect mechanisms.

**Figure 1:**
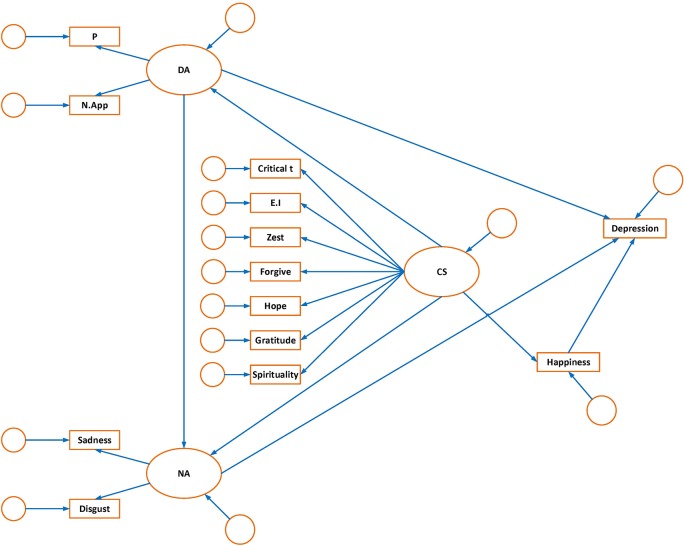
Hypothesized model of depression:measuremen t and structural components. OA, dysfunctional attitude; P, perfectionism; N.App, need for approval; NA, negative affect; Critical T, critical thinking; CS, character strength; E. I, emotional intelligence.

The main aim of the present study was to formulate and test a model of depression that considered dysfunctional attitudes, basic emotions, and character strengths. The purpose of the current research was threefold: (a) to develop a structural equation model based on theoretical understandings, (b) to examine the relationship between the constructs that predict depression, and (c) to examine the general compatibility of the model. The proposed model is shown in Figure 1.

The proposed model includes three latent variables (dysfunctional attitudes, basic negative affect, and character strengths) and one observed variable (happiness), which are identified as important in the development of depression. The model made the following a priori formulation:

1. Perfectionism and need for approval constitute the latent variable of dysfunctional attitudes. Dysfunctional attitudes would directly predict depression (Weissman and Beck, 1978; Beck, 2012). Dysfunctional attitudes would also lead individuals to experience negative emotions (Kuiper et al., 1988).
2. The two negative emotions of sadness and disgust are paired and coupled to cause depression (Power and Dalgleish, 2008). All of these emotion variables constitute the latent variable of negative affect. Negative affect would also directly lead to depression (Watson et al., 1988).
3. Some character strengths (critical thinking, hope, vitality, emotional intelligence, gratitude, forgiveness, spirituality) were selected as a result of a literature review which highlighted the most effective character strengths to overcome depression. These character strengths constitute the latent variable of character strengths. We hypothesized that a decrease in character strengths can directly lead to higher levels of dysfunctional attitudes and negative affect. Dysfunctional attitudes and negative affect, in turn, influence depression. Lower levels of character strengths can also decrease happiness and low happiness can increase depression. Therefore, character strengths would indirectly affect depression.

## Materials and method

### Participants

This study was of a correlational design. All participants had major depressive disorder (MDD) and completed a set of measures that assessed demographic variables, depression, dysfunctional attitudes, experienced emotions, and character strengths. Inclusion criteria for the participants included having a diagnosis of major depression based on DSM-5 criteria, and at least 18 years of age. Participants were excluded if they met criteria for any other DSM-5 diagnosis. The participants included 61 (30.5%) men and 139 (69.5%) women, with a mean age of 29.71 (*SD* = 7.76; range = 18–50). 88% of the participants were married. All participants were undergoing clinical interviews in preparation for psychotherapy.

### Materials

A series of questionnaires were administered which aimed to measure the constructs depicted in the hypothesized model. The latent constructs included were negative affect, character strength, and dysfunctional attitudes in addition to two measured constructs, depression, and happiness.

### Basic emotions scale (BES)

This scale was developed by Power (2006) and is composed of five subscales; anger, sadness, disgust, anxiety, and happiness. The scale is composed of 20 items (four items for each subscale) and each item is rated on a seven point Likert scale ranging from 1 (never) to 7 (very often). Each subscale has a summed score ranging from 4 to 28. Higher scores in each subscale indicate a higher intensity of emotional experience. The BES was translated into Persian and was shown to have good internal consistencies (Tehranchi et al., in press). The internal consistencies of the subscales varied from 0.79 to 0.84 in the previous research (Power, 2006). Confirmatory factor analysis indicated that a five factor model that allowed for the factors to interrelate was the best fitting model in both samples of students and individuals with depression and anxiety (Power, 2006; Power and Tarsia, 2007). Three subscales of sadness, disgust, and happiness were used in present study. Cronbach's alphas for the subscales ranged from 0.50 to 0.79 in the present study.

### Beck depression inventory-II (BDI-II)

The BDI-II is the revised version of a self-report measure of depression (Beck et al., 1996). This measure consists of 21 items. Each item is rated on a 4 point Likert scale (0–3) with a total score ranging from 0 to 63. Higher scores on the BDI-II indicate higher levels of depression severity. The BDI-II was translated into Persian and has been showed to have good internal consistency (α = 0.87) and test-retest reliability (*r* = 0.74) (Ghassemzadeh et al., 2005). Internal consistency of this measure was satisfactory (α = 0.87) in the present study.

### Dysfunctional attitudes scale-40 form a (DAS-40)

The DAS-A is a self-report scale designed to measure dysfunctional attitudes composed of 40 items. This measure has two subscales and one total score. The two subscales are perfectionism and need for approval. Internal consistency and test-retest reliability for the DAS-40 and all its subscales have been shown to be satisfactory (e.g., Oliver and Baumgart, 1985; Cane et al., 1986). The subscale of perfectionism has a summed score ranging from 15 to 105 and the subscale of need to approval has a summed score ranging from 11 to 77. Higher scores indicate more dysfunctional attitudes. The Persian version of the DAS-A has also demonstrated good internal consistency and reliability (Ebrahimi, 2009). In the present study, internal consistencies for the two subscales of perfectionism and need for approval were 0.81 and 0.87, respectively.

### Values in action-inventory of strengths (VIA-IS-240)

This is a self-report inventory developed by Peterson and Seligman (2004) to examine 24 character strengths. Items are rated on a Likert scale ranging from 1 (not at all like me) to 5 (very much like me). The 240-item questionnaire was selected to be used in the current study. This questionnaire covers 24 character strengths and 10 items are allocated to each character strength. The total score of each subscale ranges from 10 to 70. Higher scores indicate higher levels of character strengths. Average scores were used to compute character strengths. Different studies have found good reliability and validity for this inventory (Littman-Ovada and Lavy, 2012; McGrath, 2014). The seven character strengths of hope, vitality, critical thinking, emotional intelligence, forgiveness, gratitude, and spirituality were selected for the present research. In the current study, Cronbach's alphas ranged between 0.79 and 0.88.

### Procedure

The participants were recruited from patients who were referred to clinical psychology centers in the city of Mashhad (Iran). These patients had received a diagnosis of major depressive disorder based on DSM-5 criteria. Participation was voluntary and consent was obtained prior to involvement in the study. Once consent was obtained, a member of the research team would conduct a clinical interview to ensure the participant met inclusion criteria. A package of questionnaires was then given to the participants to complete. In total, completing the questionnaires took 45–60 min.

## Results

SPSS-23 and AMOS-23 was used to analyze the data. There were some missing values in the data; however, they were relatively small and randomly distributed (<1.0%). Appropriate group means were used to replace the missing values.

Means and standard deviations for the 13 measured variables examined in this study are shown in Table 1. Zero-order correlations between the variables are presented in Table 2.

**Table 1:** Possible score ranges, means and standard deviations of the variables.

**Variable**	**Possible range**	**M**	***SD***
Depression	0–63	31.95	10.79
**CHARACTER STRENGTH**			
Critical thinking	10–70	37.78	6.65
Emotional intelligence	10–70	34.12	6.97
Zest	10–70	32.21	8.04
Forgiveness	10–70	34.68	7.84
Hope	10–70	30.67	7.37
Gratitude	10–70	40.74	5.23
Spirituality	10–70	35.51	7.82
**NEGATIVE AFFECT**			
Sadness	4–28	18.30	4.95
Anger	4–28	17.57	4.12
Disgust	4–28	15.40	5.96
**DYSFUNCTIONAL ATTITUDES**			
Perfectionism	15–105	57.79	15.53
Need for approval	11–77	44.21	11.39
Happiness	4–28	14.10	5.00

**Table 2:** Zero–order correlations between the variables.

	**1**	**2**	**3**	**4**	**5**	**6**	**7**	**8**	**9**	**10**	**11**	**12**	**13**
1.BDI	1												
2.Critical thinking	−0.25**	1											
3.Emotional intelligence	−0.16*	0.51**	1										
4.Zest	−0.27**	0.54**	0.56**	1									
5.Forgiveness	−0.17*	0.20**	0.18*	0.29**	1								
6.Hope	−0.23**	0.47**	0.50**	0.57**	0.37**	1							
7.Gratitude	−0.16*	0.44**	0.37**	0.39**	0.39**	0.50**	1						
8.Spirituality	−0.26**	0.20**	0.09	0.30**	0.27**	0.31**	0.43**	1					
9.Perfectionism	0.39**	−0.26*	−0.16*	−0.20**	−0.14*	−0.25**	−0.27**	−0.36**	1				
10.Need to approval	0.39**	−0.24**	−0.14*	−0.24**	−0.16*	−0.35**	−0.22**	−0.33**	0.60**	1			
11.Sadness	0.54**	−0.35**	−0.16*	−0.29**	−0.14*	−0.38**	−0.14*	−0.29**	0.40**	0.38**	1		
12.Disgust	0.50**	−0.32**	−0.16*	−0.20**	−0.15*	−0.27**	−0.14*	−0.15*	0.34**	0.33**	0.52**	1	
13.Happiness	−0.42**	0.20**	0.27**	−0.31**	0.18*	0.27**	0.24**	0.14*	−0.29**	−0.22**	−0.38**	−36**	1

* p < 0.05;

** *p < 0.01*.

Analysis of the correlations revealed that there existed significant positive relationships between all character strengths and happiness. Similarly, all dysfunctional attitudes were positively associated with depression and negative emotions. Character strengths, displayed significant negative correlations with depression, negative emotions, and dysfunctional attitudes. In total, all obtained correlations were consistent with the findings in the empirical literature.

### Structural equation modeling (SEM)

Estimates of adequate sample size for SEM vary among researchers. Quintana and Maxwell (1999) have suggested that statistical indices generally perform adequately and yield meaningful values when there are at least 200 participants. Other researchers suggest having at least 10 participants per estimated parameter is acceptable for SEM (Schreiber et al., 2006). Thus using this criteria, the sample size of the present study was adequate. The relationship between measured variables and latent constructs in the hypothesized model are schematically portrayed in Figure 1. The hypothesized model was tested with the maximum likelihood method in AMOS-23 statistical software. Several goodness of fit indices were used to evaluate how well the structural equation models fitted with the data. Chi square as one of the most common goodness of fit indexes was significant in the hypothesized model (*X*^2^ = 186.0; *df* = 60; *p* < 0.001). This suggests that the fitness of the data to the hypothesized model was less than adequate. However, large sample theory is a prerequisite for structural equation modeling and finding well-fitted hypothesized models that have chi square values close to degrees of freedom has been proven to be unlikely (Ullman, 2001). Normed or comparative chi-square is another index that is less sensitive to sample size. It is computed through dividing chi-square by the degrees of freedom. Whilst the criterion that is considered acceptable varies among researchers, a value <5 is generally considered permissible (Schumacker and Lomax, 2004). In this study, the value of normed chi-square was 3.09 for the hypothesized model. Two other fit indices were computed as they are less sensitive to sample size. These were incremental fit index (IFI) and comparative fit index (CFI). Values for both IFI and CFI range from 0 to 1. In general, the values of 0.9 and above are regarded as acceptable. However, other researchers have suggested more stringent values of 0.95 or above as being indicative of a well-fitting model (Hu and Bentler, 1995).

In this study the IFI and CFI were computed to be 0.86 and 0.85 respectively. This suggests that the data displayed an inadequate fit with the model. In addition, the issue of model parsimony was examined with other indexes including parsimony CFI. It has been suggested that the value of 0.5 and above is representative of a well-fitting model. In this study, the value of PCFI was computed to be 0.66, suggesting an adequate fit to the model. Root Mean Square Error of Approximation (RMSEA) is another fit index. Values less than 0.08 are acceptable (Browne and Cudeck, 1993), but ideally values should be <0.05 (Steiger, 1990). The value of this index was computed to be 0.1 for the hypothesized model indicating that there was a less than adequate value for the fitness of model.

**Figure 2:**
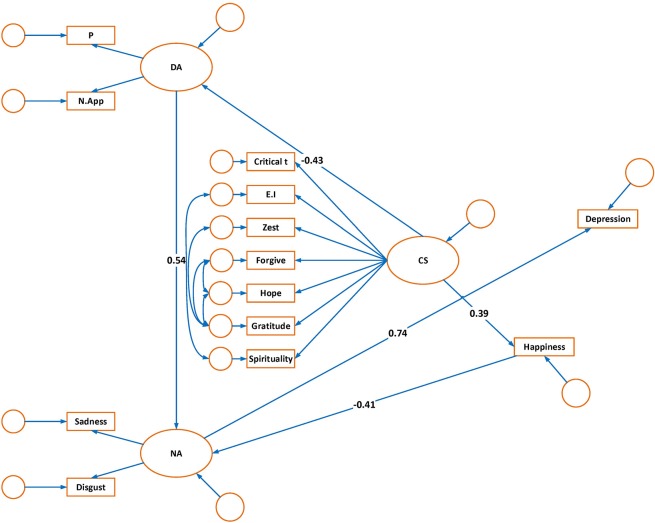
Modified model of depression:structural path coefficients.

Martens (2005) pointed out that model modifications can be made as long as they are based on theoretical and empirical criteria. In order to have a better fitting model, *post hoc* model modification were performed and critical ratios were examined to eliminate weak paths. Modification indices (MI) were also used to add paths in the model. Three structural paths were found to have a critical ratio < ±1.96 suggesting that these paths could be eliminated from the model: (a) dysfunctional attitudes to depression, (b) happiness to depression and (c) character strengths to negative affect. In addition, the MI suggested adding one structural path from happiness to negative affect. Five co-variances among residual errors of the character strengths were added as they were suggested by the MI. This empirical guidance can also be supported by theoretical reasons. According to Power and Dalgleish (2008), two basic emotions of disgust and sadness are coupled to cause depression. So, it seems theoretically plausible that negative affect has the only strong path to depression whilst other variables have indirect effects on depression through mediation of negative affect. Furthermore, the goal of positive psychology is the cultivation of character strengths and virtues to increase human wellbeing (Seligman, 2012). Positive emotions are considered as the constituents of wellbeing (Seligman, 2012); however, negative emotions mostly contribute to psychopathology. Previous research has also demonstrated that character strengths had stronger and more consistent relationship with wellbeing while vulnerabilities had stronger and more consistent relationship with illbeing (Huta and Hawley, 2010). Nevertheless, the direct effect of character strengths on the negative constructs (dysfunctional attitudes and negative affect) was hypothesized in the proposed model because lack or excess of character strengths are formulated in the psychopathology of mental disorders (Rashid, 2014). The findings demonstrated that one direct path (character strengths to dysfunctional attitudes) was supported, but another one should be eliminated. Following these adjustments, the findings indicated an adequate fit of the model to the data (*X*^2^ = 126.9, *df* = 57; Normed *X*^2^ = 2.22; CFI = 0.92; IFI = 0.92; PCFI = 0.67; RMSEA = 0.07). Furthermore, the parameter estimates for all structural paths in the modified model were statistically significant. The schematic presentation of the modified model is presented in Figure 2.

The standardized and unstandardized maximum likelihood estimates are shown in Table 2. According to the model, depression was directly predicted by the variable of negative affect. The structural path coefficient for the variable of negative affect was 0.74, indicating that high negative affect had a direct impact on depression. Dysfunctional attitudes were also positively related to negative affect (0.54). The relationship between dysfunctional attitudes and depression was found to be mediated by negative affect (standardized indirect effect = 0.40). Character strengths were negatively related to dysfunctional attitudes (−0.43). However, they were positively related to happiness (0.39). The relationship between character strengths and depression was mediated by dysfunctional attitudes, negative affect, and happiness (standardized indirect effect = −0.29).

Happiness was also negatively related to negative affect (−0.41). Therefore, happiness had an indirect effect on depression through the mediation of negative affect (standardized indirect effect = −0.30).

The squared multiple correlation coefficients are presented in Table 3. They indicate the proportion of the variance that is explained by the predictors for each variable in question. As depressed mood and guilt are two major symptoms of depression, it was expected that negative affect explain a high percentage of depression. Consistent with this hypothesis, 54% of the variance in depression was predicted by negative affect.

**Table 3:** Maximum likelihood estimates for the modified model.

**Path**	**Unstandardized**	**Standardized**		**Critical**
	**estimate**	**estimate**	**SE**	**ratio**
Character Strengths 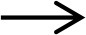 Happiness	0.601	0.385	0.153	3.939
Character Strengths 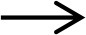 Dysfunctional Attitudes	−1.666	−0.432	0.436	−3.820
Dysfunctional Attitudes 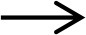 Negative Affect	0.174	0.542	0.030	5.738
Happiness 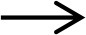 Negative Affect	−0.322	−0.406	0.059	−5.485
Negative affect 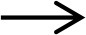 Depression	1.978	0.737	0.244	8.112

*All critical ratios had significant paths*.

Negative affect was predicted by dysfunctional attitudes and happiness, 53% of the variance in negative affect was accounted for by these two predictors. Dysfunctional attitudes was predicted by character strengths with 18% of the variance in dysfunctional attitudes being accounted for by this predictor. Happiness was predicted by character strengths with 18% of the variance in happiness accounted for by this predictor (Table 4).

**Table 4:** Squared multiple correlations (SMCs) for all the Endogenous variables in modified model.

**Predictor to endogenous variable**	**SMC**
Negative affect	0.532
Dysfunctional attitudes	0.186
Happiness	0.184
Depression	0.544

*Happiness and depression are measured variables. Negative affect and dysfunctional attitudes are latent variables*.

## Discussion

The current study was set out to examine a model of depression considering both vulnerabilities and capabilities. Dysfunctional attitudes were not found to have a direct influence on depression; however, they had a strong influence on negative affect. Negative affect was the only variable that had a direct influence on depression. In contrast, dysfunctional attitudes displayed indirect effects on depression through the mediation of negative affect. These findings are consistent with previous research that highlight the role of the two negative emotions of sadness and disgust in the etiology of depression (Power and Tarsia, 2007). That only 53% of negative affect was explained by the proposed model suggests that negative emotions were not only generated through dysfunctional attitudes but also through other factors.

Power and Dalgleish (1997, 2008) in their SPAARS^1^ model proposed two ways of generating emotions. The first way, is that some emotions are generated via schematic model. This way of generating emotions is consistent with the post-cognitive emotion model. Another way is through the associative pathway. This pathway articulates how emotions can be generated without the existence of appraisals in the time of events' occurrence (Power and Dalgleish, 2008). These emotions are generated through the appraisals that occur in the emotional history of an individual's experience of events. Thus, when emotions are generated associatively, it appears as if a concurrent process of appraisal is happening even though it is not and in fact it has occurred in the emotional past (Power and Dalgleish, 2008). Developments in affective neuroscience have also indicated that emotional processing of simple sensory features occur early in the processing sequence (Le Doux, 1996; Forgas, 2000). In order to generate an emotion, information is transferred through the thalamus to the amygdala up to the cortex (Le Doux, 1996). Information is predominantly processed in the amygdala before a person can recognize and understand the emotions consciously (Greenberg and Watson, 2006). Le Doux (1996) concluded that there are two ways to generate emotions. One is called the “low road” based on the role of the amygdala in the generation of emotions, the other, is called the “high road” and is a slower process. Information in the second way is transferred from the thalamus to the neocortex where emotions are generated through a more detailed analysis.

The findings of the present study also demonstrated that happiness had a direct influence on negative affect. This suggests that happiness has an independent role in increasing resilience or decreasing the effects of negative emotions on depression. In a similar vein, previous research has indicated a positive relationship between positive emotions and resiliency, an important component for understanding depression (Greenberg and Watson, 2006). Fredrickson (2001) has demonstrated the adaptive functions of positive emotions. Positive emotions facilitate problem solving by helping individuals to have more flexible, creative, and efficient thoughts and plans. Positive emotions can increase resilience toward depression via nullifying the effects of negative emotions on depression (Fredrickson, 2001). The neuropsychological approach of Davidson (2000) also highlighted how a stable positive affective style developed resilience to depression whereas a low positive affective style increased an individual's vulnerability to experiencing depression.

Another finding of this study was a strong negative correlation between happiness and depressive symptoms. Previous research demonstrated that positive emotions were associated with depressive symptoms among European Americans but not among Asian immigrants in America (Leu et al., 2011). The largest Asian ethnic groups in that study were Chinese, Korean, and Vietnamese (Leu et al., 2011). Given the findings of the present study, it seems that the role of positive emotions among the Iranian individuals with depression is more similar to the western population than to the Asian population.

Seven character strengths of hope, critical thinking, emotional intelligence, gratitude, forgiveness, spirituality, and zest were selected in the current research as the strengths that were hypothesized to be the most influential in the etiology of depression. Character strengths were found to have a strong effect on the two variables of dysfunctional attitudes and happiness. Thus, character strengths displayed an indirect effect on depression through the mediation of dysfunctional attitudes and happiness. This finding is consistent with previous research which did not find any significant relationships between character strengths and depression among clinically depressed individuals (Huta and Hawley, 2010). However, it has been shown that pre-treatment character strengths are related to post-treatment recovery from depression (Huta and Hawley, 2010). Consistent with previous research, the findings of present study demonstrated a positive relationship between character strengths and positive emotions (Littman-Ovada and Lavy, 2012; Azañedoa et al., 2014; Martínez-Martí and Ruch, 2014).

The predictive effects of character strengths on dysfunctional attitudes suggests that individuals with low levels of character strengths are vulnerable to the generation of dysfunctional attitudes. The current findings suggest that some character strengths like critical thinking may help an individual to recognize and reduce any dysfunctional attitudes that they may hold. Furthermore, emotional intelligence may also help people to become aware of their own and others emotional experiences and use such awareness to assist in solving problems and regulating emotions (Salovey and Mayer, 1990).

We suggest some character strengths may support the development of positive and adaptive attitudes toward the self. The character strengths like emotional intelligence, zest, hope, gratitude, forgiveness, and spirituality can increase the agency of self. They can help an individual to consider himself as strong and effective; consequently, such a person doesn't need to generate dysfunctional attitudes to compensate for the weakness of self. Indeed, two dysfunctional attitudes of need for approval and perfectionism can be considered as the ways to defend the weakness of the self.

Although it is beyond the scope of the current study, the findings of the proposed model may have some implications for practitioners to focus on negative emotions especially sadness and disgust when treating depression. The findings suggest that in order to regulate negative emotions, dysfunctional attitudes should be challenged and some character strengths should be cultivated. As the most variance of negative affect was not explained by dysfunctional attitudes, cognitive techniques are not sufficient to change negative emotions in depression. Usual techniques in CBT such as identifying and challenging negative thoughts do not work properly when emotions are generated associatively. This implies that emotion-focused techniques should be used more frequently in the treatment of depression. In these techniques, emotion memories are brought into awareness so that they can be symbolized in the conscious awareness and be exposed to new emotional experience (Greenberg and Watson, 2006). When clients label experiences stored in their implicit memory and they become aware of their maladaptive emotions, they can differentiate between past and present events and be better able to identify their needs in their current situations (Greenberg and Watson, 2006).

### Limitations and strengths of the present study

Although the current study provides evidence in support of the proposed model of depression, it does have several limitations. First, the proposed model did not explain the majority of variances in depression; that is, other third variables are likely influential in depression, which were not considered in our model. Our model also had a limited number of variables as the participants would be unable to complete so many questionnaires. However, the present study did shed light on the relationship between character strengths and some cognitive and emotional factors related to depression. Moreover, there was an imbalance in marital status and gender of the participants that may affect the generalizability of the study. Nonetheless, such imbalance may represent the preponderance of married women with mood disorders in Iran as previous research has also demonstrated that mood disorders are associated with female gender and being married in an Iranian population (Mohammadi et al., 2006). Third, the sample size in our study is not large. Replication of this study in a larger sample size could enhance the generalizability of the model. Forthly, self-report questionnaires might be insufficient to measure character strengths. It is recommended to include other instruments such as interviews to measure character strengths in future research. It is also recommended that researchers use the findings of the present study to develop an integrative model for the treatment of depression.

Considering both vulnerabilities and capabilities in the psychopathology of depression is one of the major strengths of the present study. The findings complement previous psychopathology models that only focus on negative or positive factors associated with depression. Another positive point of the current study was selecting character strengths based on the psychopathology of depression. Additionally, the findings of this study gives a more nuanced understanding in the relationship between character strengths and vulnerabilities.

## Conclusion

In conclusion, the structural equation modeling analyses demonstrated an adequate fit between the proposed model and the data. Clinically, the structural paths between the predictor variables and the dependent variable in the proposed model appeared to be consistent with our hypotheses, though required some modifications. It should be emphasized that this was an observational study and causality cannot be inferred. Furthermore, structural equation modeling has been described as a combination of exploratory factor analysis and multiple regressions (Ullman, 2001). Thus, the findings of the study indicate some predictive effects that can be considered in the psychopathology and treatment of depression. The proposed model serves to identify the role of character strengths on vulnerabilities such as dysfunctional attitudes and negative affect.

## Ethics statement

This study was carried out in accordance with the recommendations of Institutional Review Board at University of Isfahan with written informed consent from all subjects. All subjects gave written informed consent in accordance with the Declaration of Helsinki. The protocol was approved by the ethical committee of the University of Isfahan.

## Author contributions

The contribution of AT was in data collection, data analysis, and writing the paper. The contribution of MP was in data analysis and writing the paper. The contributions of SA and HN were in writing the paper.

### Conflict of interest statement

The authors declare that the research was conducted in the absence of any commercial or financial relationships that could be construed as a potential conflict of interest.

## Footnotes

^1^Schematic Propositional Associative Analogical Representing Systems

## References

[B1] American Psychiatric Association (2013). Diagnostic and Statistical Manual of Mental Disorders, 5th Edn. Washington, DC: American Psychiatric Association.

[B2] Azañedoa C. M. Fernández-Abascalb E. G. Barracac J. (2014). Character strengths in Spain: validation of the values in action inventory of strengths (VIA-IS) in a Spanish sample. Clínica. Y. Salud. 25, 123–130. 10.1016/j.clysa.2014.06.002

[B3] Bannink F. P. (2014). Positive CBT: from reducing distress to building success. J. Contemp. Psychother. 44, 1–8. 10.1007/s10879-013-9239-7

[B4] Beck A. T. Steer R. A. Brown G. K. (1996). Manual for the Beck Depression Inventory-II. San Antonio, TX: Psychological Corporation.

[B5] Beck J. S. (2012). Cognitive Behavior Therapy: Basics and Beyond 2nd Edn. New York, NY: the Guilford Press.

[B6] Browne M. W. Cudeck R. (1993). Alternative ways of assessing model fit in Testing Structural Equation Models, eds Bollen K. A. Long J. S. (Newsbury Park, CA: Sage), 136–162.

[B7] Cane D. B. Olinger J. Gotlib I. H. Kuiper N. A. (1986). Factor structure of the Dysfunctional Attitude Scale in a student population. J. Clin. Psychol. 42, 307–309. 10.1002/1097-4679(198603)42:2<307::AID-JCLP2270420213>3.0.CO;2-J

[B8] Chao R. C.-L. (2015). Counseling Psychology: An Integrated Positive Psychological Approach. Chichester, UK: Wiley-Blackwell.

[B9] Danhauer S. C. Legault C. Bandos H. Kidwell K. Constantino J. Vaughan L.. (2013). Positive and negative affect, depression, and cognitive processes in the cognition in the study of tamoxifen and raloxifene (Co-STAR) trial. Neuropsychol. Dev. Cogn. B Aging Neuropsychol. Cogn. 20, 1–18. 10.1080/13825585.2012.747671 23237718 PMC3815441

[B10] Davidson R. (2000). Affective style, mood and anxiety disorders: An affective neuroscience approach in Anxiety, Depression and Emotion, eds Davidson R. (Oxford: Oxford University Press), 23–55.

[B11] Disabato D. J. Short J. L. Kashdan T. B. Curby T. W. Jarden A. (2014). Do character strengths reduce future depression or does depression reduce character strengths? Present Am Psychol Assoc. 10.1037/e542342014-001

[B12] Doolittle B. R. Farrell M. (2004). the association between spirituality and depression in an urban clinic. Prim. Care Companion J. Clin. Psychiatry. 6, 114–118. 10.4088/PCC.v06n0302 15361925 PMC474734

[B13] Downey L. A. Johnston P. J. Hansen K. Schembri R. Stough C. Tuckwell V. (2008). The relationship between emotional intelligence and depression in a clinical sample. Eur. J. Psychiatry 22, 93–98. 10.4321/S0213-61632008000200005

[B14] Ebrahimi A. (2009). Effectiveness of Integrated Treatment of Religious, Cognitive Behavioral and Drug Therapy on Depression and Dysfunctional Attitudes of Patients With Dysthymic Disorder. Ph.D. unpublished doctoral dissertation, University of Isfahan, Isfahan.

[B15] Esmaeili Z. Bagheri M. (2015). Evaluation of the relationship between critical thinking skills and affective control in child training students of the female technical and vocational college in the city of Broujerd. J. Educ. Pract. 6, 28–36.

[B16] Forgas J. P. (2000). Feeling is believing? The role of processing strategies in mediating affective influences on beliefs in Emotions and Belief: How Feelings Influence Thoughts. Studies in Emotion and Social Interaction, eds Frijda N. H. Manstead A. S. R. Bern S. (New York, NY: Cambridge University Press), 108–143.

[B17] Fredrickson B. L. (2001). The role of positive emotions in positive psychology: the broaden-and-build theory of positive emotions. Am. Psychol. 56, 218-−226. 10.1037//0003-066X.56.3.218 11315248 PMC3122271

[B18] Fredrickson B. L. (2004). Gratitude, Like Other Positive Emotions, Broadens and Builds in Series in Affective Science. The Psychology of Gratitude, eds Emmons R. A. McCullough M. E. (New York, NY: Oxford University Press), 145–166.

[B19] Freidlin P. Littman-Ovadia H. Niemiec R. M. (2017). Positive psychopathology: social anxiety via character strengths underuse and overuse. Pers. Individ. Dif. 108, 50–54. 10.1016/j.paid.2016.12.003

[B20] Ghassemzadeh H. Mojtabai R. Karamghadiri N. Ebrahimkhani N. (2005). Psychometric properties of Persian-language version of the Beck Depression Inventory-Second edition: BDI-II-Persian. Depress. Anxiety 21, 185–192. 10.1002/da.20070 16075452

[B21] Gillham J. Adams-Deutsch Z. Werner J. K. Reivich K. Coulter-Heindl V. Linkins M. (2011). Character strengths predict subjective well-being during adolescence J. Posit. Psychol. 6, 31–44. 10.1080/17439760.2010.536773

[B22] Greenberg L. S. (2002). Emotion-Focused Therapy: Coaching Clients to Work Through Their Feelings. Washington, DC: American Psychological Association.

[B23] Greenberg L. S. Watson J. C. (2006). Emotion-Focused Therapy for Depression Washington, DC: American Psychological Association.

[B24] Hodgson L. K. Wertheim E. K. (2007). Does good emotion management aid forgiving? Multiple dimensions of empathy, emotion management and forgiveness of self and others. J. Soc. Pers. Relat. 24, 931–949. 10.1177/0265407507084191

[B25] Hu L. T. Bentler P. M. (1995). Evaluating model fit in Structural Equation Modeling: Concepts, Issues, and Applications, eds Hoyle R. H. (Thousand Oaks, CA: Sage), 76–99.

[B26] Huta V. Hawley L. (2010). Psychological strengths and cognitive vulnerabilities: are they two ends of the same continuum or do they have independent relationships with well-being and ill-being? J. Happiness Stud. 11, 71–93. 10.1007/s10902-008-9123-4

[B27] Koenig H. G. McCollough M. E. Larson D. B. (2001). Handbook of Religion and Health. London: Oxford University Press.

[B28] Kuiper N. A. Olinger L. J. Martin R. A. (1988). Dysfunctional attitudes, stress and negative emotions. Cognit. Ther. Res. 12, 533–547. 10.1007/BF01205008

[B29] Le Doux J. (1996). The Emotional Brain: The Mysterious Underpinnings of Emotional Life. New York, NY: Simon &. Schuster.

[B30] Leu J. Wang J. Koo K. (2011). Are positive emotions just as “positive” across cultures? Emotion 11, 994–999. 10.1037/a0021332 21443338

[B31] Lim N. (2016). Cultural differences in emotion: differences in emotional arousal level between the East and the West. Integr. Med. Res. 5, 105–109. 10.1016/j.imr.2016.03.004 28462104 PMC5381435

[B32] Littman-Ovada H. Lavy S. (2012). Character Strengths in Israel: hebrew adaptation of the VIA inventory of strengths. Euro. J. Psychol. Assess. 28, 41–50. 10.1027/1015-5759/a000089

[B33] Luna N. MacMillan T. (2015). The relationship between spirituality and depressive symptom severity, psychosocial functioning impairment, and quality of life: examining the impact of age, gender, and ethnic differences. Ment. Health Relig. Cult. 18, 513–525. 10.1080/13674676.2015.1087481

[B34] Martens M. P. (2005). The use of structural equation modeling in counseling psychology research. Couns. Psychol. 33, 269–298. 10.1177/0011000004272260

[B35] Martínez-Martí M. L. Ruch W. (2014). Character strengths and well-being across the life span: data from a representative sample of German-speaking adults living in Switzerland. Front. Psychol. 5:1253. 10.3389/fpsyg.2014.01253 25408678 PMC4219388

[B36] McGrath R. E. (2014). Integrating psychological and cultural perspectives on virtue: the hierarchical structure of character strengths. J. Posit. Psychol. 10, 407–424. 10.1080/17439760.2014.994222

[B37] Meyer J. H. McMain S. Kennedy S. H. Korman L. Brown G. M. DaSilva J. N.. (2003). Dysfunctional attitudes and 5-HT2 receptors during depression and self-harm. Am. J. Psychiatry 160, 90–99. 10.1176/appi.ajp.160.1.90 12505806

[B38] Miranda J. Persons J. B. (1988). Dysfunctional attitudes are mood-state dependent. J. Abnorm. Psychol. 97, 76–79. 10.1037/0021-843X.97.1.76 3351115

[B39] Mohammadi M. R. Ghanizadeh A. Davidian H. Malekafzali H. Naghavi H. R. Pouretemad H. R. (2006). Prevalence of mood disorders in Iran. Iran. J. Psychiatry. 1, 59–64.

[B40] Oliver J. M. Baumgart E. P. (1985). The dysfunctional attitudes scale: psychometric properties and relation to depression in an unselected adult population. Cogn. Ther. Res. 9, 161–167. 10.1007/BF01204847

[B41] Park C. L. (2007). Religiousness/spirituality and health: a meaning systems perspective. J. Behav. Med. 30, 319–328. 10.1007/s10865-007-9111-x 17522971

[B42] Peterson C. Seligman M. P. (2004). Character Strengths and Virtues: A Handbook and Classification. New York, NY: Oxford University Press Washington, DC: American Psychological Association.

[B43] Power M. (2010). Emotion-Focused Cognitive Therapy. Chichester, UK: A John Wily & Sons publications Ltd.

[B44] Power M. J. (2006). The structure of emotion: an empirical comparison of six models. Cogn. Emot. 20, 694–713. 10.1080/02699930500367925

[B45] Power M. J. Dalgleish T. (1997). Cognition and Emotion: From Order to Disorder. Hove: Psychology Press.

[B46] Power M. J. Dalgleish T. (2008). Cognition and Emotion: From Order to Disorder, 2nd Edn. Hove: Erlbaum.

[B47] Power M. J. Tarsia M. (2007). Basic and complex emotions in depression and anxiety. Clin. Psychol. Psychother. 14, 19–31. 10.1002/cpp.515

[B48] Quintana S. M. Maxwell S. E. (1999). Implications of recent developments in structural equation modeling for counseling psychology. Couns. Psychol. 27, 485–527.

[B49] Rashid T. (2014). Positive psychotherapy: a strength-based approach. J. Posit. Psychol. 10, 25–40. 10.1080/17439760.2014.920411

[B50] Ross R. Smith G. R. Booth B. M. (1997). Treatment outcomes in depressed patients. Psychiatr. Ann. 27, 119–123. 10.3928/0048-5713-19970201-13

[B51] Sahin N. H. Sahin N. (1992). How dysfunctional are the dysfunctional attitudes in another culture? Psychol. Psychother. 65, 17–26. 10.1111/j.2044-8341.1992.tb01680.x 1571303

[B52] Salovey P. Mayer J. D. (1990). Emotional intelligence. Imagin. Cogn. Pers. 9, 185–211. 10.2190/DUGG-P24E-52WK-6CDG

[B53] Schreiber J. B. Nora A. Stage F. K. Barlow E. A. King J. (2006). Reporting structural equation modeling and confirmatory factor analysis results: a review. J. Educ. Res. 99, 323–337. 10.3200/JOER.99.6.323-338

[B54] Schumacker R. E. Lomax R. G. (2004). A Beginner's Guide to Structural Equation Modeling, 2nd Edn. Mahwah, NJ: Lawrence Erlbaum Associates.

[B55] Seligman M. E. P. (2012). Flourish: A New Visionary Understanding of Happiness. New York, NY: Free Press.

[B56] Steiger J. H. (1990). Structural model evaluation and modification: an interval estimation approach. Multivariate Behav. Res. 25, 173–180. 10.1207/s15327906mbr2502_4 26794479

[B57] Teasdale J. D. (1988). Cognitive vulnerability to persistent depression. Cogn. Emot. 2, 247–274. 10.1080/02699938808410927

[B58] Thimm J. C. Holte A. Brennen T. Wang C. E. (2013). Hope and expectancies for future events in depression. Front. Psychol. 4:470. 10.3389/fpsyg.2013.00470 23898314 PMC3721024

[B59] Toussaint L. L. Williams D. R. Musick M. A. Everson-Rose S. A. (2008). Why forgiveness may protect against depression: helplessness as an exploratory mechanism. Personal. Ment. Health. 2, 89–103. 10.1002/pmh.35

[B60] Ullman J. B. (2001). Structural equation modeling in Using Multivariate Statistics, 4th Edn, eds Tabachnick B. G. Fidell L. S. (Needham Heights, MA: Allyn and Bacon), 676–684.

[B61] Watson D. Clark L. A. Carey G. (1988). Positive and negative affectivity and their relation to anxiety and depressive disorders. J. Abnorm. Psychol. 97, 346–353. 10.1037/0021-843X.97.3.346 3192830

[B62] Weissman A. N. Beck A. T. (1978). Development and validation of dysfunctional attitudes scale: a preliminary investigation in Paper Presented at the Annual Meeting of the American Educational Research Association (Toronto, ON).

[B63] Wong P. T. P. (2010). What is existential positive psychology? Int. J. Existent. Psychol. Psychother. 3, 1–10.

[B64] Wood A. M. Maltby J. Gillett R. Linley P. A. Joseph S. (2008). the role of gratitude in the development of social support, stress, and depression. J. Res. Pers. 42, 854–871. 10.1016/j.jrp.2007.11.003

[B65] Wood A. M. Tarrier N. (2010). positive clinical psychology: a new vision and strategy for integrated research and practice. Clin. Psychol. Rev. 30, 819–882. 10.1016/j.cpr.2010.06.003 20655136

[B66] Zajonc R. B. (2000). Feeling and thinking: Closing the debate over the independence of affect in Feeling and Thinking: The Role of Affect in Social Cognition, eds Forgas J. P. (New York, NY: Cambridge University Press), 31–58.

